# Supplementary material for: Mo-Doped Na4Fe3(PO4)2P2O7/C Composites for High-Rate and Long-Life Sodium-Ion Batteries
Source: Materials (Basel). 2024 Jun 1;17(11):2679. doi: 10.3390/ma17112679 (PMC11174099; doi:10.3390/ma17112679)
Supplement: Supplementary file 1 [file materials-17-02679-s001.zip › materials-3012023-supplementary.pdf]

Supplementary Information for

Mo-doped $Na_4Fe_3(PO_4)_2P_2O_7$/C Composites for High Rate and Long-life Sodium-ion Batteries

Tongtong Chen [1,2], Xianying Han [1,2], Mengling Jie [1,2], Zhiwu Guo [1,2], Jiangang Li [1,2,*] and Xiangming He [3,*]

[1] College of New Materials and Chemical Engineering, Beijing Institute of Petrochemical Technology,
Beijing 102617, China; ctt10105204@163.com (T.C.); hanxianying@bipt.edu.cn (X.H.);
jml16953708@163.com (M.J.); guozhiwu@bipt.edu.cn (Z.G.)

[2] Beijing Key Laboratory of Fuels Cleaning and Advanced Catalytic Emission Reduction Technology,
Beijing 102617, China

[3] Institute of Nuclear & New Energy Technology, Tsinghua University, Beijing 100084, China

* Correspondence: lijiangang@bipt.edu.cn (J.L.); hexm@tsinghua.edu.cn (X.H.)

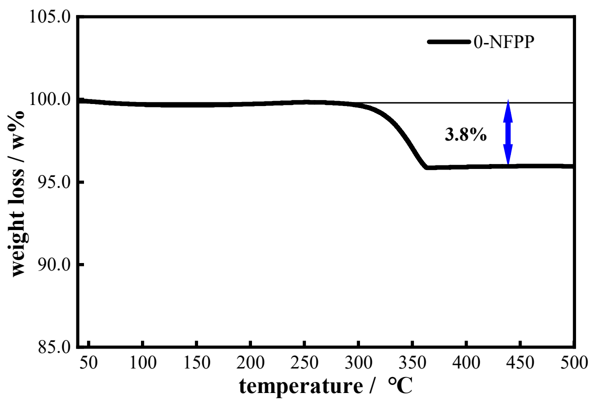

Figure S1 Thermogravimetric diagrams of 0-NFPP.

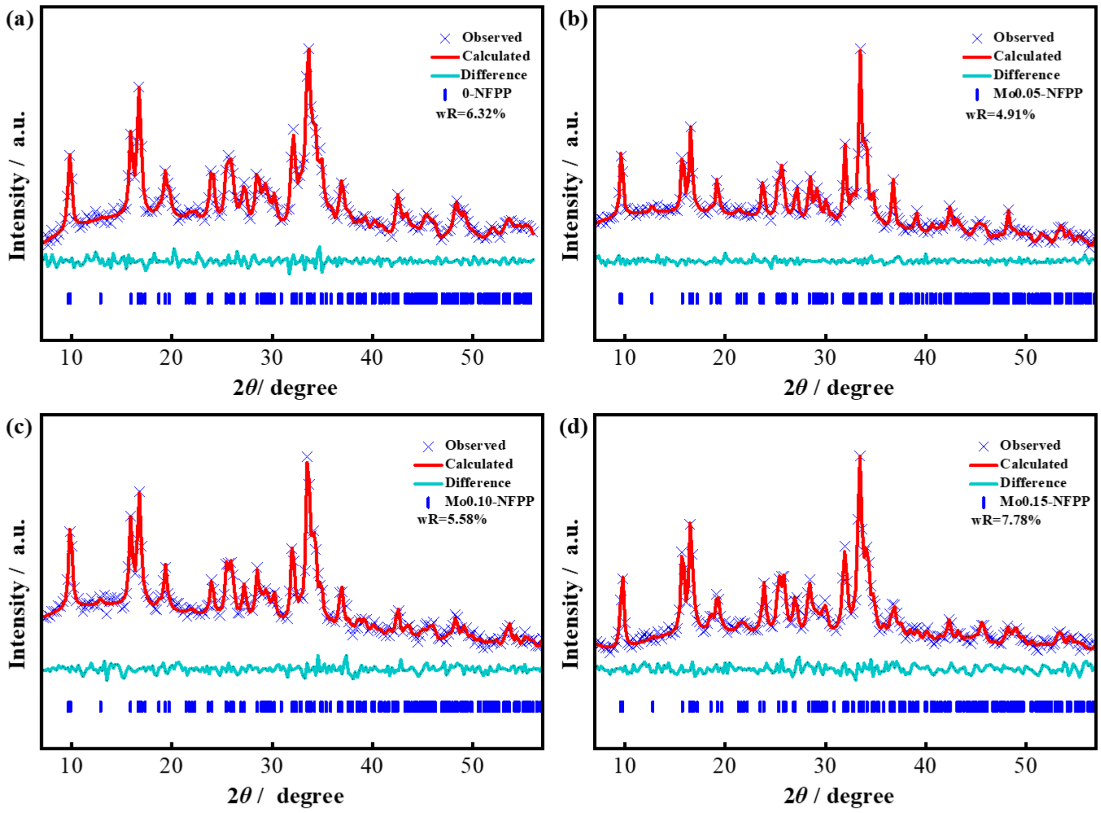

Figure S2 Rietveld refinements of the XRD patterns of (a) 0-NFPP, (b) Mo0.05-NFPP, (c) Mo0.10-NFPP, and (d) Mo0.15-NFPP samples.

Table S1 Lattice parameters of Mox-NFPP materials.

**Sample**	**0-NFPP**	**Mo0.05-NFPP**	**Mo0.10-NFPP**	**Mo0.15-NFPP**
a (Å)	18.038751	18.102355	18.055107	18.015246
b (Å)	6.555948	6.555246	6.569176	6.590088
c (Å)	10.706099	10.678863	10.714705	10.706736
V ($Å^3$)	1266.15	1267.212	1270.841	1271.126
wR	6.32%	4.91%	5.58%	7.78%

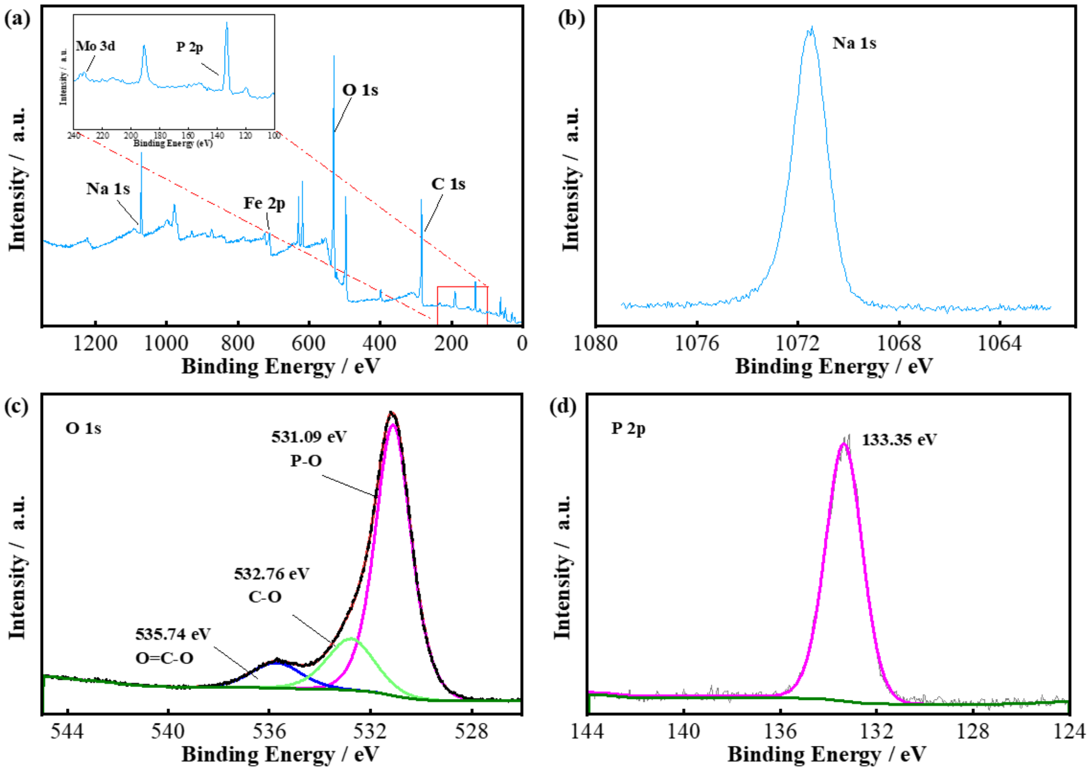

Figure S3 (a) XPS survey, and (b) Na 1s, (c) O 1s, (d) P 2p spectra of Mo0.10-NFPP.

Table S2 Impedance parameters of Mox-NFPP materials

**Sample**	**0-NFPP**	**Mo0.05-NFPP**	**Mo0.10-NFPP**	**Mo0.15-NFPP**
R_s (Ω)	6.50	5.60	4.50	4.00
R_{ct} (Ω)	1485	1253	725	1144
W (Ω $s^{-1/2}$)	182	261	310	312
CPE	300 nF	300 nF	300 nF	300 nF
	α=0.82	α=0.81	α=0.80	α=0.82

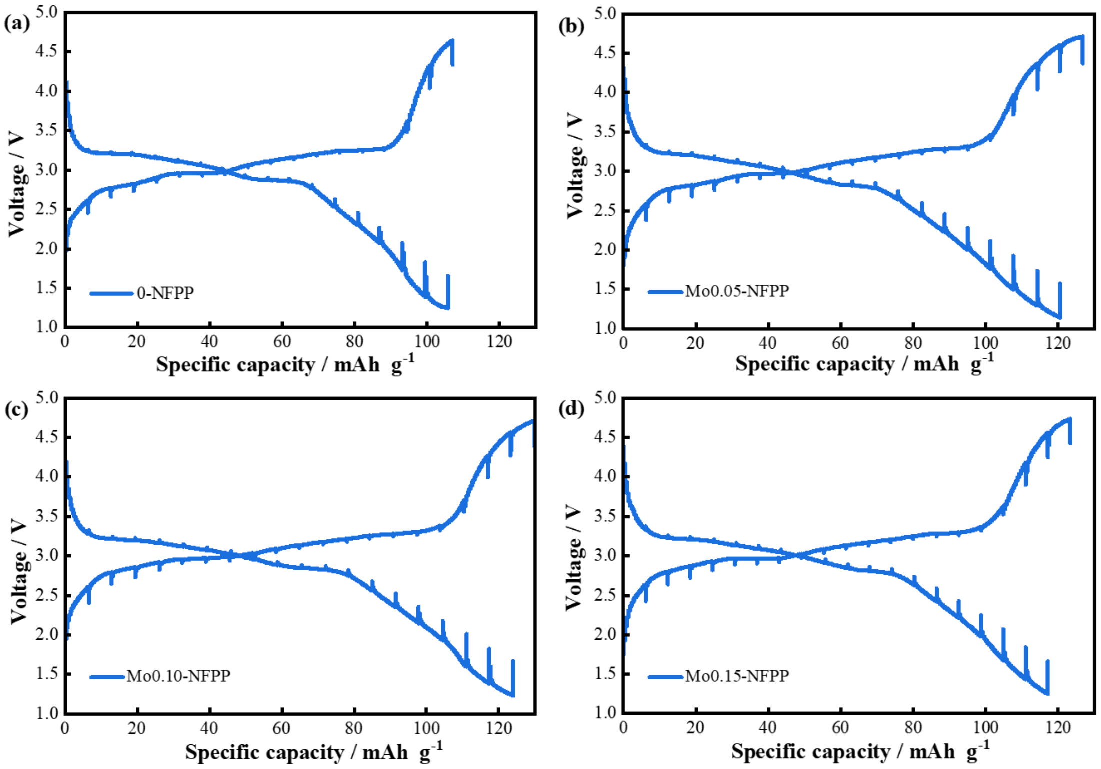

Figure S4 GITT curves of (a) 0-NFPP, (b) Mo0.05-NFPP, (c) Mo0.10-NFPP, (d) Mo0.15-NFPP.

Table S3 Comparison of electrochemical performance of ions doped NFPP materials from different research.

Doped ions	Conductive coating materials	Discharge capacity (mAh g^{-1})	Capacity retention	Ref.
Mo^{6+}	C	123.9 (0.1C)<br>95.71 (5C)<br>86.09 (10C)	96.18% (500 cycles, 10C)	This work
Mn^{2+}	rGO	131.5 (0.1C)<br>97.3 (5C)<br>90.8 (10C)	91.6% (200 cycles, 2C)<br>96.7% (700 cycles, 5C)<br>97.2 (2000 cycles, 10C)	[S1]
Mn^{2+}	C	119.6 (0.1C)<br>88.97 (5C)<br>81.66 (10C)	94.6% (1000 cycles, 10C)	[S2]
Mg^{2+}	C	104 (0.05 A g^{-1})<br>~75 (5 A g^{-1})	90.4% (5000 cycles, 5 A g^{-1})	[S3]
Cr^{3+}	C<br>CNT	105.44 (0.1C)<br>89 (5C)<br>120.64 (0.1C)<br>87.11 (20C)	91.97% (500 cycles, 10C)<br>92.37% (2000 cycles, 10C)	[S4]
Ti^{4+}	C	105.3 (0.2C)<br>84.9 (5C)<br>80 (10C)	91.4% (200 cycles, 1C)<br>93.8% (2000 cycles, 10C)	[S5]
V^{3+}	C	123.4 (0.1C)<br>105.7 (5C)<br>102.3 (10C)	81.65% (10000 cycles, 20C)	[S6]

References:

[S1] Li, X.Q.; Zhang, Y.; Zhang, B.L.; Qin, K.; Liu, H.M.; Ma, Z.F. Mn-doped $Na_4Fe_3(PO_4)_2(P_2O_7)$ facilitating Na^+ migration at low temperature as a high performance cathode material of sodium ion batteries. *J. Power Sources* **2022**, *521*, 230922-230933.

[S2] Tao, Q.D.; Ding, H.Y.; Tang, X.; Zhang, K.B.; Teng, J.H.; Zhao, H.M.; Li, J. Mn-doped $Na_4Fe_3(PO_4)_2P_2O_7$ as a low-cost and high-performance cathode material for sodium-ion batteries. *Energy Fuels* **2023**, *37*, 6230−6239.

[S3] Xiong, F.Y.; Li, J.T.; Zuo, C.L.; Zhang, X.L.; Tan, S.S.; Jiang, Y.L.; An, Q.Y.; Chu, P.K.; Mai, L.Q. Mg-doped $Na_4Fe_3(PO_4)_2(P_2O_7)/C$ composite with enhanced intercalation pseudocapacitance for ultra-stable and high-rate sodium-ion storage. *Adv. Funct. Mater.* **2023**, *33*: 2211257-2211265.

[S4] Wang, G.H.; Chen T.T.; Chen J.; Zhang Z.Y.; Zhen C.; Han X.Y.; Li J.G. Preparation and electrochemical properties of $Na_4Fe_{3-x}Cr_x(PO_4)_2P_2O_7/C@CNT$ cathode materials for sodium ion batteries. *Mod. Chem. Ind.* **2024**, *44(5)*, 149-154.

[S5] Huang, L.; Liu, C.J.; Bao, L.; Chen, Y.; Jiang, Y.H.; Fu, X.C. Large scalable preparation of Ti-doped $Na_4Fe_3(PO_4)_2P_2O_7$ as cathode material for high rate and long-life sodium-ion batteries. *ACS Appl. Energ. Mater.* **2023**, *6*, 11541-11549.

[S6] Zhang, H.; Cao, Y.J.; Liu, Z.L.; Cheng, X.S.; Li, X.L.; Xu, J.; Wang, N.; Yang, H.; Liu, Y.; Zhang, J.X. Structurally modulated $Na_{4-x}Fe_{3-x}V_x(PO_4)_2P_2O_7$ by vanadium doping for long-life sodium-ion batteries. *ACS Sustainable Chem. Eng.* **2024**, *12*, 5310−5318.
